# Quantum Programming with Inductive Datatypes: Causality and Affine Type Theory

**DOI:** 10.1007/978-3-030-45231-5_29

**Published:** 2020-04-17

**Authors:** Romain Péchoux, Simon Perdrix, Mathys Rennela, Vladimir Zamdzhiev

**Affiliations:** 8 ENS/CNRS, Université Paris-Saclay, Cachan, France; 9 University of Duisburg-Essen, Duisburg, Germany; 10 Université de Lorraine, CNRS, Inria, LORIA, 54000 Nancy, France; 11 Leiden University, Leiden, The Netherlands

**Keywords:** Quantum programming, Inductive types, Adequacy

## Abstract

Inductive datatypes in programming languages allow users to define useful data structures such as natural numbers, lists, trees, and others. In this paper we show how inductive datatypes may be added to the quantum programming language QPL. We construct a sound categorical model for the language and by doing so we provide the first detailed semantic treatment of user-defined inductive datatypes in quantum programming. We also show our denotational interpretation is invariant with respect to big-step reduction, thereby establishing another novel result for quantum programming. Compared to classical programming, this property is considerably more difficult to prove and we demonstrate its usefulness by showing how it immediately implies computational adequacy at all types. To further cement our results, our semantics is entirely based on a physically natural model of von Neumann algebras, which are mathematical structures used by physicists to study quantum mechanics.

### 1 Introduction

*Quantum computing* is a computational paradigm which takes advantage of quantum mechanical phenomena to perform computation. A quantum computer can solve problems which are out of reach for classical computers (e.g. factorisation of large numbers [24], solving large linear systems [8]). The recent developments of quantum technologies points out the necessity of filling the gap between theoretical quantum algorithms and the actual (prototypes of) quantum computers. As a consequence, quantum software and in particular quantum programming languages play a key role in the future development of quantum computing. The present paper makes several theoretical contributions towards the design and denotational semantics of quantum programming languages.

Our development is based around the quantum programming language QPL [23] which we extend with inductive datatypes. Our paper is the first to construct a denotational semantics for user-defined inductive datatypes in quantum programming. In the spirit of the original QPL, our type system is *affine* (discarding of arbitrary variables is allowed, but copying is restricted). We also extend QPL with a copy operation for *classical data*, because this is an admissible operation in quantum mechanics which improves programming convenience. The addition of inductive datatypes requires a departure from the original denotational semantics of QPL, which are based on finite-dimensional quantum structures, and we consider instead (possibly infinite-dimensional) quantum structures based on *W*-algebras* (also known as *von Neumann algebras*), which have been used by physicists in the study of quantum foundations [25]. As such, our semantic treatment is physically natural and our model is more accessible to physicists and experts in quantum computing compared to most other denotational models.

QPL is a first-order programming language which has *procedures*, but it does not have lambda abstractions. Thus, there is no use for a !-modality and we show how to model the copy operation by describing the canonical comonoid structure of all classical types (including the inductive ones).

An important notion in quantum mechanics is the idea of *causality* which has been formulated in a variety of different ways. In this paper, we consider a simple operational interpretation of causality: if the output of a physical process is discarded, then it does not matter which process occurred [10]. In a symmetric monoidal category $\mathbf{C}$ with tensor unit $I$, this can be understood as requiring that for any morphism (process) $f : A_1 \to A_2$, it must be the case that $\diamond_{A_2} \circ f = \diamond_{A_1}$, where $\diamond_{A_i} : A_i \to I$ is the discarding map (process) at the given objects. This notion ties in very nicely with our affine language, because we have to show that the interpretation of values is causal, i.e., values are always discardable.

A major contribution of this paper is that we prove the denotational semantics is invariant with respect to both small-step reduction and big-step reduction. The latter is more difficult in quantum programming and our paper is the first to demonstrate such a result. As a corollary, we obtain computational adequacy.

### 2 Syntax of QPL

The syntax of QPL (including our extensions) is summarised in Figure 1. A well-formed type context, denoted $\vdash \Theta$, is simply a list of distinct type variables. A type $A$ is well-formed in type context $\Theta$, denoted $\Theta \vdash A$, if the judgement can be derived according to the following rules (see [1,6] for a more detailed exposition):

$$\frac{\vdash \Theta}{\Theta \vdash \Theta_i} \qquad \frac{\vdash \Theta}{\Theta \vdash I} \qquad \frac{\vdash \Theta}{\Theta \vdash \mathbf{qbit}} \qquad \frac{\Theta \vdash A \qquad \Theta \vdash B}{\Theta \vdash A \star B} \star \in \{+, \otimes\} \qquad \frac{\Theta, X \vdash A}{\Theta \vdash \mu X.A}$$

A type $A$ is *closed* if $\cdot \vdash A$. Note that nested type induction is allowed. Henceforth, we implicitly assume that all types we are dealing with are well-formed.

*Example 1.* The type of natural numbers is defined as $\mathbf{Nat} \equiv \mu X.I + X$. Lists of a closed type $\cdot \vdash A$ are defined as $\mathbf{List}(A) \equiv \mu Y.I + A \otimes Y$.

Notice that our type system is not equipped with a !-modality. Indeed, in the absence of function types, there is no reason to introduce it. Instead, we specify

| | | |
|---|---|---|
| Types | $A, B$ | $::= X \mid I \mid \mathbf{qbit} \mid A + B \mid A \otimes B \mid \mu X.A$ |
| Classical Types | $P, R$ | $::= X \mid I \mid P + R \mid P \otimes R \mid \mu X.P$ |
| Terms | $M, N$ | $::= \mathbf{new\ unit}\ u \mid \mathbf{discard}\ x \mid y = \mathbf{copy}\ x \mid \mathbf{new\ qbit}\ q \mid$ $b = \mathbf{measure}\ q \mid q_1, \dots, q_n \ {*}{=}\ S \mid M; N \mid \mathbf{skip} \mid$ $\mathbf{while}\ b\ \mathbf{do}\ M \mid x = \mathbf{left}_{A,B} M \mid x = \mathbf{right}_{A,B} M \mid$ $\mathbf{case}\ y\ \mathbf{of}\ \{\mathbf{left}\ x_1 \to M \mid \mathbf{right}\ x_2 \to N\} \mid$ $x = (x_1, x_2) \mid (x_1, x_2) = x \mid y = \mathbf{fold}\ x \mid y = \mathbf{unfold}\ x \mid$ $\mathbf{proc}\ f :: \ x : A \to y : B\ \{M\} \ \mid y = f(x)$ |
| Variable contexts | $\Gamma, \Sigma$ | $::= x_1 : A_1, \dots, x_n : A_n$ |
| Procedure contexts | $\Pi$ | $::= f_1 : A_1 \to B_1, \dots, f_n : A_n \to B_n$ |

$$\frac{}{\Pi \vdash \langle \Gamma \rangle\ \mathbf{new\ unit}\ u\ \langle \Gamma, u : I \rangle} \qquad \frac{}{\Pi \vdash \langle \Gamma, x : A \rangle\ \mathbf{discard}\ x\ \langle \Gamma \rangle}$$

$$\frac{P \text{ is a classical type}}{\Pi \vdash \langle \Gamma, x : P \rangle\ y = \mathbf{copy}\ x\ \langle \Gamma, x : P, y : P \rangle} \qquad \frac{}{\Pi \vdash \langle \Gamma \rangle\ \mathbf{skip}\ \langle \Gamma \rangle}$$

$$\frac{\Pi \vdash \langle \Gamma \rangle\ M\ \langle \Gamma' \rangle \qquad \Pi \vdash \langle \Gamma' \rangle\ N\ \langle \Sigma \rangle}{\Pi \vdash \langle \Gamma \rangle\ M; N\ \langle \Sigma \rangle}$$

$$\frac{\Pi \vdash \langle \Gamma, b : \mathbf{bit} \rangle\ M\ \langle \Gamma, b : \mathbf{bit} \rangle}{\Pi \vdash \langle \Gamma, b : \mathbf{bit} \rangle\ \mathbf{while}\ b\ \mathbf{do}\ M\ \langle \Gamma, b : \mathbf{bit} \rangle}$$

$$\frac{}{\Pi \vdash \langle \Gamma \rangle\ \mathbf{new\ qbit}\ q\ \langle \Gamma, q : \mathbf{qbit} \rangle} \qquad \frac{}{\Pi \vdash \langle \Gamma, q : \mathbf{qbit} \rangle\ b = \mathbf{measure}\ q\ \langle \Gamma, b : \mathbf{bit} \rangle}$$

$$\frac{S \text{ is a unitary of arity } n}{\Pi \vdash \langle \Gamma, q_1 : \mathbf{qbit}, \dots, q_n : \mathbf{qbit} \rangle\ q_1, \dots, q_n \ {*}{=}\ S\ \langle \Gamma, q_1 : \mathbf{qbit}, \dots, q_n : \mathbf{qbit} \rangle}$$

$$\frac{}{\Pi \vdash \langle \Gamma, x : A \rangle\ y = \mathbf{left}_{A,B}\ x\ \langle \Gamma, y : A + B \rangle}$$

$$\frac{}{\Pi \vdash \langle \Gamma, x : B \rangle\ y = \mathbf{right}_{A,B}\ x\ \langle \Gamma, y : A + B \rangle}$$

$$\frac{\Pi \vdash \langle \Gamma, x_1 : A \rangle\ M_1\ \langle \Sigma \rangle \qquad \Pi \vdash \langle \Gamma, x_2 : B \rangle\ M_2\ \langle \Sigma \rangle}{\Pi \vdash \langle \Gamma, y : A + B \rangle\ \mathbf{case}\ y\ \mathbf{of}\ \{\mathbf{left}_{A,B}\ x_1 \to M_1 \mid \mathbf{right}_{A,B}\ x_2 \to M_2\ \}\ \langle \Sigma \rangle}$$

$$\frac{}{\Pi \vdash \langle \Gamma, x_1 : A, x_2 : B \rangle\ x = (x_1, x_2)\ \langle \Gamma, x : A \otimes B \rangle}$$

$$\frac{}{\Pi \vdash \langle \Gamma, x : A \otimes B \rangle\ (x_1, x_2) = x\ \langle \Gamma, x_1 : A, x_2 : B \rangle}$$

$$\frac{}{\Pi \vdash \langle \Gamma, x : A[\mu X.A/X] \rangle\ y = \mathbf{fold}_{\mu X.A}\ x\ \langle \Gamma, y : \mu X.A \rangle}$$

$$\frac{}{\Pi \vdash \langle \Gamma, x : \mu X.A \rangle\ y = \mathbf{unfold}\ x\ \langle \Gamma, y : A[\mu X.A/X] \rangle}$$

$$\frac{\Pi, f : A \to B \vdash \langle x : A \rangle\ M\ \langle y : B \rangle}{\Pi \vdash \langle \Gamma \rangle\ \mathbf{proc}\ f :: \ x : A \to y : B\ \{M\}\ \langle \Gamma \rangle}$$

$$\frac{}{\Pi, f : A \to B \vdash \langle \Gamma, x : A \rangle\ y = f(x)\ \langle \Gamma, y : B \rangle}$$

Fig. 1: Syntax and formation rules for QPL terms.

the subset of types where copying is an admissible operation. The *classical types* are a subset of our types defined in Figure 1. They are characterised by the property that variables of classical types may be copied, whereas variables of non-classical types may not be copied (see the rule for copying in Figure 1).

We use small Latin letters (e.g. $x, y, u, q, b$) to range over *term variables*. More specifically, $q$ ranges over variables of type **qbit**, $u$ over variables of unit type $I$, $b$ over variables of type $\mathbf{bit} := I + I$ and $x, y$ range over variables of arbitrary type. We use $\Gamma$ and $\Sigma$ to range over *variable contexts*. A variable context is a function from term variables to *closed types*, which we write as $\Gamma = x_1 : A_1, \ldots, x_n : A_n$.

We use $f, g$ to range over *procedure names*. Every procedure name $f$ has an *input type* $A$ and an *output type* $B$, denoted $f : A \to B$, where $A$ and $B$ are closed types. We use $\Pi$ to range over *procedure contexts*. A procedure context is a function from procedure names to pairs of procedure input-output types, denoted $\Pi = f_1 : A_1 \to B_1, \ldots, f_n : A_n \to B_n$.

*Remark 2.* Unlike lambda abstractions, procedures cannot be passed to other procedures as input arguments, nor can they be returned as output.

A *term judgement* has the form $\Pi \vdash \langle \Gamma \rangle\ M\ \langle \Sigma \rangle$ (see Figure 1) and indicates that term $M$ is well-formed in procedure context $\Pi$ with input variable context $\Gamma$ and output variable context $\Sigma$. All types occurring within it are closed.

The intended interpretation of the quantum rules are as follows. The term **new qbit** $q$ prepares a new qubit $q$ in state $|0\rangle\langle 0|$. The term $q_1, \ldots, q_n \mathrel{*}= S$ applies a unitary operator $S$ to a sequence of qubits in the standard way. The term $b = \mathbf{measure}\ q$ performs a quantum measurement on qubit $q$ and stores the measurement outcome in bit $b$. The measured qubit is destroyed in the process.

The no-cloning theorem of quantum mechanics [28] shows that arbitrary qubits cannot be copied. Because of this, copying is restricted only to classical types, as indicated in Figure 1, and this allows us to avoid runtime errors. Like the original QPL [23], our type system is also *affine* and so any variable can be discarded (see the formation rule for the term **discard** $x$ in Figure 1).

### 3 Operational Semantics of QPL

In this section we describe the operational semantics of QPL. The central notion is that of a *program configuration* which provides a complete description of the current state of program execution. It consists of four components that must satisfy some coherence properties: (1) the term which remains to be executed; (2) a *value assignment*, which is a function that assigns formal expressions to variables as a result of execution; (3) a *procedure store* which keeps track of what procedures have been defined so far and (4) the *quantum state* computed so far.

*Value Assignments.* A *value* is an expression defined by the following grammar:

$$v, w ::= * \mid n \mid \mathbf{left}_{A,B} v \mid \mathbf{right}_{A,B} v \mid (v, w) \mid \mathbf{fold}_{\mu X.A} v$$

where $n$ ranges over the natural numbers. Think of $*$ as representing the unique value of unit type $I$ and of $n$ as representing a pointer to the $n$-th qubit of a quantum state $\rho$. Specific values of interest are $\texttt{ff} := \textbf{left}_{I,I}*$ and $\texttt{tt} := \textbf{right}_{I,I}*$ which correspond to **false** and **true** respectively.

A *qubit pointer context* is a set $Q$ of natural numbers. A value $v$ of type $A$ is well-formed in qubit pointer context $Q$, denoted $Q \vdash v : A$, if the judgement is derivable from the following rules:

$$\frac{}{\cdot \vdash * : I} \qquad \frac{}{\{n\} \vdash n : \textbf{qbit}} \qquad \frac{Q \vdash v : A}{Q \vdash \textbf{left}_{A,B} v : A + B} \qquad \frac{Q \vdash v : B}{Q \vdash \textbf{right}_{A,B} v : A + B}$$

$$\frac{Q_1 \vdash v : A \qquad Q_2 \vdash w : B \qquad Q_1 \cap Q_2 = \varnothing}{Q_1, Q_2 \vdash (v, w) : A \otimes B} \qquad \frac{Q \vdash v : A[\mu X.A/X]}{Q \vdash \textbf{fold}_{\mu X.A} v : \mu X.A}$$

If $v$ is well-formed, then its type and qubit pointer context are uniquely determined. If $Q \vdash v : P$ with $P$ classical, then we say $v$ is a *classical value.*

**Lemma 3.** *If $Q \vdash v : P$ is a well-formed classical value, then $Q = \cdot$.*

A *value assignment* is a function from term variables to values, which we write as $V = \{x_1 = v_1, \ldots, x_n = v_n\}$, where $x_i$ are variables and $v_i$ are values. A value assignment is *well-formed* in qubit pointer context $Q$ and variable context $\Gamma$, denoted $Q; \Gamma \vdash V$, if $V$ has exactly the same variables as $\Gamma$, so that $\Gamma = \{x_1 : A_1, \ldots, x_n : A_n\}$, and $Q = Q_1, \ldots, Q_n$, s.t. $Q_i \vdash v_i : A_i$. Such a splitting of $Q$ is necessarily unique, if it exists, and some of the $Q_i$ may be empty.

*Procedure Stores.* A *procedure store* is a set of procedure definitions, written as:

$$\Omega = \{f_1 :: x_1 : A_1 \to y_1 : B_1 \ \{M_1\}, \ldots, f_n :: x_n : A_n \to y_n : B_n \ \{M_n\}\}\,.$$

A procedure store is *well-formed* in procedure context $\Pi$, written $\Pi \vdash \Omega$, if the judgement is derivable via the following rules:

$$\frac{}{\cdot \vdash \cdot} \qquad \frac{\Pi \vdash \Omega \qquad \Pi, f : A \to B \vdash \langle x : A \rangle \ M \ \langle y : B \rangle}{\Pi, f : A \to B \vdash \Omega, f :: x : A \to y : B \ \{M\}}$$

*Program Configurations.* A *program configuration* is a quadruple $(M \mid V \mid \Omega \mid \rho)$, where $M$ is a term, $V$ is a value assignment, $\Omega$ is a procedure store and $\rho \in \mathbb{C}^{2^n \times 2^n}$ is a finite-dimensional density matrix with $0 \leq \mathrm{tr}(\rho) \leq 1$. The density matrix $\rho$ represents a (mixed) quantum state and its trace may be smaller than one because we also use it to encode probability information (see Remark 4). We write $dim(\rho) = n$ to indicate that the dimension of $\rho$ is $n$.

A *well-formed* program configuration is a configuration $(M \mid V \mid \Omega \mid \rho)$, where there exist (necessarily unique) $\Pi, \Gamma, \Sigma, Q$, such that: (1) $\Pi \vdash \langle \Gamma \rangle \ M \ \langle \Sigma \rangle$ is a well-formed term; (2) $Q; \Gamma \vdash V$ is a well-formed value assignment; (3) $\Pi \vdash \Omega$ is a well-formed procedure store; and (4) $Q = \{1, 2, \ldots, dim(\rho)\}$. We write $\Pi; \Gamma; \Sigma; Q \vdash (M \mid V \mid \Omega \mid \rho)$ to indicate this situation. The formation rules enforce that the qubits of $\rho$ and the qubit pointers from $V$ are in a 1-1 correspondence.

$$\frac{}{(\textbf{new unit } u \mid V \mid \Omega \mid \rho) \rightsquigarrow (\textbf{skip} \mid V, u = * \mid \Omega \mid \rho)}$$

$$\frac{}{(\textbf{discard } x \mid V, x = v \mid \Omega \mid \rho) \rightsquigarrow (\textbf{skip} \mid r_v(V) \mid \Omega \mid tr_v(\rho))}$$

$$\frac{}{(y = \textbf{copy } x \mid V, x = v \mid \Omega \mid \rho) \rightsquigarrow (\textbf{skip} \mid V, x = v, y = v \mid \Omega \mid \rho)}$$

$$\frac{}{(\textbf{new qbit } q \mid V \mid \Omega \mid \rho) \rightsquigarrow (\textbf{skip} \mid V, q = dim(\rho) + 1 \mid \Omega \mid \rho \otimes |0\rangle\langle 0|)}$$

$$\frac{}{(\vec{q} \mathrel{*}= S \mid V, \vec{q} = \vec{m} \mid \Omega \mid \rho) \rightsquigarrow (\textbf{skip} \mid V, \vec{q} = \vec{m} \mid \Omega \mid S_{\vec{m}}(\rho))}$$

$$\frac{}{(b = \textbf{measure } q \mid V, q = m \mid \Omega \mid \rho) \rightsquigarrow (\textbf{skip} \mid r_m(V), b = \texttt{ff} \mid \Omega \mid {}_m\langle 0|\rho|0\rangle_m)}$$

$$\frac{}{(b = \textbf{measure } q \mid V, q = m \mid \Omega \mid \rho) \rightsquigarrow (\textbf{skip} \mid r_m(V), b = \texttt{tt} \mid \Omega \mid {}_m\langle 1|\rho|1\rangle_m)}$$

$$\frac{}{(\textbf{skip}; P \mid V \mid \Omega \mid \rho) \rightsquigarrow (P \mid V \mid \Omega \mid \rho)} \qquad \frac{(P \mid V \mid \Omega \mid \rho) \rightsquigarrow (P' \mid V' \mid \Omega' \mid \rho')}{(P; Q \mid V \mid \Omega \mid \rho) \rightsquigarrow (P'; Q \mid V' \mid \Omega' \mid \rho')}$$

$$\frac{}{(\textbf{while } b \textbf{ do } M \mid V, b = \texttt{ff} \mid \Omega \mid \rho) \rightsquigarrow (\textbf{skip} \mid V, b = \texttt{ff} \mid \Omega \mid \rho)}$$

$$\frac{}{(\textbf{while } b \textbf{ do } M \mid V, b = \texttt{tt} \mid \Omega \mid \rho) \rightsquigarrow (M; \textbf{while } b \textbf{ do } M \mid V, b = \texttt{tt} \mid \Omega \mid \rho)}$$

$$\frac{}{(y = \textbf{left } x \mid V, x = v \mid \Omega \mid \rho) \rightsquigarrow (\textbf{skip} \mid V, y = \textbf{left } v \mid \Omega \mid \rho)}$$

$$\frac{}{(y = \textbf{right } x \mid V, x = v \mid \Omega \mid \rho) \rightsquigarrow (\textbf{skip} \mid V, y = \textbf{right } v \mid \Omega \mid \rho)}$$

$$\frac{}{(\textbf{case } y \textbf{ of } \{\textbf{left } x_1 \to M_1 \mid \textbf{right } x_2 \to M_2\ \} \mid V, y = \textbf{left } v \mid \Omega \mid \rho) \rightsquigarrow (M_1 \mid V, x_1 = v \mid \Omega \mid \rho)}$$

$$\frac{}{(\textbf{case } y \textbf{ of } \{\textbf{left } x_1 \to M_1 \mid \textbf{right } x_2 \to M_2\ \} \mid V, y = \textbf{right } v \mid \Omega \mid \rho) \rightsquigarrow (M_2 \mid V, x_2 = v \mid \Omega \mid \rho)}$$

$$\frac{}{(x = (x_1, x_2) \mid V, x_1 = v_1, x_2 = v_2 \mid \Omega \mid \rho) \rightsquigarrow (\textbf{skip} \mid V, x = (v_1, v_2) \mid \Omega \mid \rho)}$$

$$\frac{}{((x_1, x_2) = x \mid V, x = (v_1, v_2) \mid \Omega \mid \rho) \rightsquigarrow (\textbf{skip} \mid V, x_1 = v_1, x_2 = v_2 \mid \Omega \mid \rho)}$$

$$\frac{}{(y = \textbf{fold } x \mid V, x = v \mid \Omega \mid \rho) \rightsquigarrow (\textbf{skip} \mid V, y = \textbf{fold } v \mid \Omega \mid \rho)}$$

$$\frac{}{(y = \textbf{unfold } x \mid V, x = \textbf{fold } v \mid \Omega \mid \rho) \rightsquigarrow (\textbf{skip} \mid V, y = v \mid \Omega \mid \rho)}$$

$$\frac{}{(\textbf{proc } f :: \ x : A \to y : B\ \{M\} \mid V \mid \Omega \mid \rho) \rightsquigarrow (\textbf{skip} \mid V \mid \Omega, f :: \ x : A \to y : B\ \{M\} \mid \rho)}$$

$$\frac{}{\begin{array}{c}(y_1 = f(x_1) \mid V, x_1 = v \mid \Omega, f :: \ x_2 : A \to y_2 : B\ \{M\} \mid \rho) \rightsquigarrow \\ (M_\alpha \mid V, x_1 = v \mid \Omega, f :: \ x_2 : A \to y_2 : B\ \{M\} \mid \rho)\end{array}}$$

Fig. 2: Small Step Operational semantics of QPL.

The small step semantics is defined for configurations $(M \mid V \mid \Omega \mid \rho)$ by induction on $M$ in Figure 2 and we now explain the notations used therein.

In the rule for discarding, we use two functions that depend on a value $v$. They are $tr_v$, which modifies the quantum state $\rho$ by tracing out all of its qubits which are used in $v$, and $r_v$ which simply reindexes the value assignment, so that the pointers within $r_v(V)$ correctly point to the corresponding qubits of $tr_v(\rho)$, which is potentially of smaller dimension than $\rho$. Formally, for a well-formed value $v$, let $Q$ and $A$ be the unique qubit pointer context and type, such that $Q \vdash v : A$. Then $tr_v(\rho)$ is the quantum state obtained from $\rho$ by tracing out all qubits specified by $Q$. Given a value assignment $V = \{x_1 = v_1, \dots x_n = v_n\}$, then $r_v(V) = \{x_1 = r'_v(v_1), \dots, x_n = r'_v(v_n)\}$, where:

$$r'_v(w) = \begin{cases} *, & \text{if } w = * \\ k - |\{i \in Q \mid i < k\}|, & \text{if } w = k \in \mathbb{N} \\ \textbf{left } r'_v(w'), & \text{if } w = \textbf{left } w' \\ \textbf{right } r'_v(w'), & \text{if } w = \textbf{right } w' \\ (r'_v(w_1), r'_v(w_2)) & \text{if } w = (w_1, w_2) \\ \textbf{fold } r'_v(w'), & \text{if } w = \textbf{fold } w' \end{cases}$$

In the rule for unitaries, the superoperator $S_{\vec{m}}$ applies the unitary $S$ to the vector of qubits specified by $\vec{m}$. In the rules for measurement, the $m$-th qubit of $\rho$ is measured in the computational basis, the measured qubit is destroyed in the process and the measurement outcome is stored in the bit $b$. More specifically, $|i\rangle_m = I_{2^{m-1}} \otimes |i\rangle \otimes I_{2^{n-m}}$ and ${}_m\langle i|$ is its adjoint, for $i \in \{0, 1\}$, and where $I_n$ is the identity matrix in $\mathbb{C}^{n \times n}$.

*Remark 4.* Because of the way we decided to handle measurements, reduction $(- \rightsquigarrow -)$ is a *nondeterministic* operation, where we encode the probabilities of reduction within the trace of our density matrices in a similar way to [9]. Equivalently, we may see the reduction relation as *probabilistic* provided that we normalise all density matrices and decorate the reductions with the appropriate probability information as specified by the Born rule of quantum mechanics. The nondeterministic view leads to a more concise and clear presentation and because of this we have chosen it over the probabilistic view.

The introduction rule for procedures simply defines a procedure which is added to the procedure store. In the rule for calling procedures, the term $M_\alpha$ is $\alpha$-equivalent to $M$ and is obtained from it by renaming the input $x_2$ to $x_1$, renaming the output $y_2$ to $y_1$ and renaming all other variables within $M$ to some fresh names, so as to avoid conflicts with the input, output and the rest of the variables within $V$.

**Theorem 5 (Subject reduction).** *If $\Pi; \Gamma; \Sigma; Q \vdash (M \mid V \mid \Omega \mid \rho)$ and $(M \mid V \mid \Omega \mid \rho) \rightsquigarrow (M' \mid V' \mid \Omega' \mid \rho')$, then $\Pi'; \Gamma'; \Sigma; Q' \vdash (M' \mid V' \mid \Omega' \mid \rho')$, for some (necessarily unique) contexts $\Pi', \Gamma', Q'$ and where $\Sigma$ is invariant.*

**Assumption 6.** *From now on we assume all configurations are well-formed.*

```
while b do {
  new qbit q;
  q *= H;
  discard b;
  b = measure q
}
```

(a) A term $M$

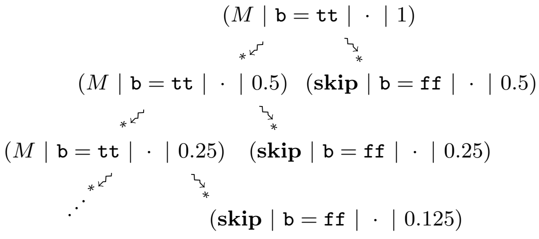

(b) A reduction graph involving $M$

Fig. 3: Example of a term and of a reduction graph.

A configuration $(M \mid V \mid \Omega \mid \rho)$ is said to be *terminal* if $M = \textbf{skip}$. Program execution finishes at terminal configurations, which are characterised by the property that they do not reduce any further. We will use calligraphic letters $(\mathcal{C}, \mathcal{D}, \ldots)$ to range over configurations and we will use $\mathcal{T}$ to range over terminal configurations. For a configuration $\mathcal{C} = (M \mid V \mid \Omega \mid \rho)$, we write for brevity $\mathrm{tr}(\mathcal{C}) := \mathrm{tr}(\rho)$ and we shall say $\mathcal{C}$ is *normalised* whenever $\mathrm{tr}(\mathcal{C}) = 1$. We say that a configuration $\mathcal{C}$ is *impossible* if $\mathrm{tr}(\mathcal{C}) = 0$ and we say it is *possible* otherwise.

**Theorem 7 (Progress).** *If $\mathcal{C}$ is a configuration, then either $\mathcal{C}$ is terminal or there exists a configuration $\mathcal{D}$, such that $\mathcal{C} \rightsquigarrow \mathcal{D}$. Moreover, if $\mathcal{C}$ is not terminal, then $\mathrm{tr}(\mathcal{C}) = \sum_{\mathcal{C} \rightsquigarrow \mathcal{D}} \mathrm{tr}(\mathcal{D})$ and there are at most two such configurations $\mathcal{D}$.*

In the situation of the above theorem, the probability of reduction is given by $\Pr(\mathcal{C} \rightsquigarrow \mathcal{D}) := \mathrm{tr}(\mathcal{D})/\mathrm{tr}(\mathcal{C})$, for any possible $\mathcal{C}$ (see Remark 4) and Theorem 7 shows the total probability of all single-step reductions is 1. If $\mathcal{C}$ is impossible, then $\mathcal{C}$ occurs with probability 0 and subsequent reductions are also impossible.

*Probability of Termination.* Given configurations $\mathcal{C}$ and $\mathcal{D}$ let $\mathrm{Seq}_n(\mathcal{C}, \mathcal{D}) := \{\mathcal{C}_0 \rightsquigarrow \cdots \rightsquigarrow \mathcal{C}_n \mid \mathcal{C}_0 = \mathcal{C} \text{ and } \mathcal{C}_n = \mathcal{D}\}$, and let $\mathrm{Seq}_{\leq n}(\mathcal{C}, \mathcal{D}) = \bigcup_{i=0}^{n} \mathrm{Seq}_n(\mathcal{C}, \mathcal{D})$. Finally, let $\mathrm{TerSeq}_{\leq n}(\mathcal{C}) := \bigcup_{\mathcal{T} \text{ terminal}} \mathrm{Seq}_{\leq n}(\mathcal{C}, \mathcal{T})$. In other words, $\mathrm{TerSeq}_{\leq n}(\mathcal{C})$ is the set of all reduction sequences from $\mathcal{C}$ which terminate in at most $n$ steps (including 0 if $\mathcal{C}$ is terminal). For every terminating reduction sequence $r = (\mathcal{C} \rightsquigarrow \cdots \rightsquigarrow \mathcal{T})$, let $\mathrm{End}(r) := \mathcal{T}$, i.e. $\mathrm{End}(r)$ is simply the (terminal) endpoint of the sequence.

For any configuration $\mathcal{C}$, the sequence $\left(\sum_{r \in \mathrm{TerSeq}_{\leq n}(\mathcal{C})} \mathrm{tr}(\mathrm{End}(r))\right)_{n \in \mathbb{N}}$ is increasing with upper bound $\mathrm{tr}(\mathcal{C})$ (follows from Theorem 7). For any possible $\mathcal{C}$, we define:

$$\mathrm{Halt}(\mathcal{C}) := \bigvee_{n=0}^{\infty} \sum_{r \in \mathrm{TerSeq}_{\leq n}(\mathcal{C})} \mathrm{tr}(\mathrm{End}(r))/\mathrm{tr}(\mathcal{C})$$

which is exactly the *probability of termination* of $\mathcal{C}$. This is justified, because $\mathrm{Halt}(\mathcal{T}) = 1$, for any terminal (and possible) configuration $\mathcal{T}$ and $\mathrm{Halt}(\mathcal{C}) = \sum_{\substack{\mathcal{C} \rightsquigarrow \mathcal{D} \\ \mathcal{D} \text{ possible}}} \Pr(\mathcal{C} \rightsquigarrow \mathcal{D}) \mathrm{Halt}(\mathcal{D})$. We write $\rightsquigarrow_*$ for the transitive closure of $\rightsquigarrow$.

```
proc GHZnext :: l : ListQ -> l : ListQ {
  new qbit q;
  case l of
      nil -> q*=H;
             l = q :: nil
    | q' :: l' -> q',q *= CNOT;
                  l = q :: q' :: l'
}

proc GHZ :: n : Nat -> l : ListQ {
  case n of
      zero -> l = nil
    | s(n') -> l = GHZnext(GHZ(n'))
}
```

(a) Procedures for generating $\mathrm{GHZ}_n$.

$$(\texttt{l = GHZ(n)} \mid \texttt{n} = \texttt{s(s(s(zero)))} \mid \Omega \mid 1)$$
$$\rightsquigarrow^*$$
$$(\texttt{l = GHZnext(l)} \mid \texttt{l} = 2 :: 1 :: \texttt{nil} \mid \Omega \mid \gamma_2)$$
$$\rightsquigarrow$$
$$(\texttt{new qbit q}; \cdots \mid \texttt{l} = 2 :: 1 :: \texttt{nil} \mid \Omega \mid \gamma_2)$$
$$\rightsquigarrow$$
$$(\texttt{case l of } \cdots \mid \texttt{l} = 2 :: 1 :: \texttt{nil}, \texttt{q} = 3 \mid \Omega \mid \gamma_2 \otimes |0\rangle\langle 0|)$$
$$\rightsquigarrow^*$$
$$(\texttt{q',q *=CNOT}; \cdots \mid \texttt{l'} = 1 :: \texttt{nil}, \texttt{q} = 3, \texttt{q'} = 2 \mid \Omega \mid \gamma_2 \otimes |0\rangle\langle 0|)$$
$$\rightsquigarrow$$
$$(\texttt{l = q :: q' :: l'} \mid \texttt{l'} = 1 :: \texttt{nil}, \texttt{q} = 3, \texttt{q'} = 2 \mid \Omega \mid \gamma_3)$$
$$\rightsquigarrow^*$$
$$(\textbf{skip} \mid \texttt{l} = 3 :: 2 :: 1 :: \texttt{nil} \mid \Omega \mid \gamma_3)$$

(b) A reduction sequence producing $\mathrm{GHZ}_3$.

Fig. 4: Example with lists of qubits and a recursive procedure.

*Example 8.* Consider the term $M$ in Figure 3. The body of the **while** loop (3a) has the effect of performing a fair coin toss (realised through quantum measurement in the standard way) and storing the outcome in variable `b`. Therefore, starting from configuration $\mathcal{C} = (M \mid \texttt{b} = \texttt{tt} \mid \cdot \mid 1)$, as in Subfigure 3b, the program has the effect of tossing a fair coin until `ff` shows up. The set of terminal configurations reachable from $\mathcal{C}$ is $\{(\textbf{skip} \mid \texttt{b} = \texttt{ff} \mid \cdot \mid 2^{-i}) \mid i \in \mathbb{N}_{\geq 1}\}$ and the last component of each configuration is a $1 \times 1$ density matrix which is exactly the probability of reducing to the configuration. Therefore $\mathrm{Halt}(\mathcal{C}) = \sum_{i=1}^{\infty} 2^{-i} = 1$.

*Example 9.* The $\mathrm{GHZ}_n$ state is defined as $\gamma_n \coloneqq (|0\rangle^{\otimes n} + |1\rangle^{\otimes n})(\langle 0|^{\otimes n} + \langle 1|^{\otimes n})/2$. In Figure 4, we define a procedure `GHZ`, which given a natural number $n$, generates the state $\gamma_n$, which is represented as a list of qubits of length $n$. The procedure (4a) uses an auxiliary procedure `GHZnext`, which given a list of qubits representing the state $\gamma_n$, returns the state $\gamma_{n+1}$ again represented as a list of qubits. The two procedures make use of some (hopefully obvious) syntactic sugar. In 4b, we also present the last few steps of a reduction sequence which produces $\gamma_3$ starting from configuration $(\texttt{l = GHZ(n)} \mid \texttt{n} = \texttt{s(s(s(zero)))} \mid \Omega \mid 1)$, where $\Omega$ contains the above mentioned procedures. In the reduction sequence we only show the term in evaluating position and we omit some intermediate steps. The type `ListQ` is a shorthand for $\textbf{List}(\textbf{qbit})$ from Example 1.

### 4 W*-algebras

In this section we describe our denotational model. It is based on W*-algebras, which are algebras of observables (i.e. physical entities), with interesting domain-theoretic properties. We recall some background on W*-algebras and their categorical structure. We refer the reader to [25] for an encyclopaedic account on W*-algebras.

*Domain-theoretic Preliminaries.* Recall that a directed subset of a poset $P$ is a non-empty subset $X \subseteq P$ in which every pair of elements of $X$ has an upper bound in $X$. A poset $P$ is a *directed-complete partial order* (*dcpo*) if each directed subset has a supremum. A poset $P$ is *pointed* if it has a least element, usually denoted by $\bot$. A monotone map $f : P \to Q$ between posets is *Scott-continuous* if it preserves suprema of directed subsets. If $P$ and $Q$ are pointed and $f$ preserves the least element, then we say $f$ is *strict*. We write **DCPO** ($\mathbf{DCPO}_{\bot!}$) for the category of (pointed) dcpo's and (strict) Scott-continuous maps between them.

*Definition of W*-algebras.* A *complex algebra* is a complex vector space $V$ equipped with a bilinear multiplication $(- \cdot -) : V \times V \to V$, which we write as juxtaposition. A *Banach algebra* $A$ is a complex algebra $A$ equipped with a submultiplicative norm $\| - \| : A \to \mathbb{R}_{\geq 0}$, i.e. $\forall x, y \in A : \|xy\| \leq \|x\|\|y\|$. A $*$-*algebra* $A$ is a complex algebra $A$ with an involution $(-)^* : A \to A$ such that $(x^*)^* = x$, $(x+y)^* = (x^* + y^*)$, $(xy)^* = y^* x^*$ and $(\lambda x)^* = \overline{\lambda} x^*$, for $x, y \in A$ and $\lambda \in \mathbb{C}$. A *C*-algebra* is a Banach $*$-algebra $A$ which satisfies the C*-identity, i.e. $\|x^* x\| = \|x\|^2$ for all $x \in A$. A C*-algebra $A$ is *unital* if it has an element $1 \in A$, such that for every $x \in A : x1 = 1x = x$. All C*-algebras in this paper are unital and for brevity we regard unitality as part of their definition.

*Example 10.* The algebra $M_n(\mathbb{C})$ of $n \times n$ complex matrices is a C*-algebra. In particular, the set of complex numbers $\mathbb{C}$ has a C*-algebra structure since $M_1(\mathbb{C}) \cong \mathbb{C}$. More generally, the $n \times n$ matrices valued in a C*-algebra $A$ also form a C*-algebra $M_n(A)$. The C*-algebra of qubits is $\mathbf{qbit} \coloneqq M_2(\mathbb{C})$.

An element $x \in A$ of a C*-algebra $A$ is called *positive* if $\exists y \in A : x = y^* y$. The *poset of positive elements* of $A$ is denoted $A^+$ and its order is given by $x \leq y$ iff $(y - x) \in A^+$. The *unit interval* of $A$ is the subposet $[0,1]_A \subseteq A^+$ of all positive elements $x$ such that $0 \leq x \leq 1$.

Let $f : A \to B$ be a linear map between C*-algebras $A$ and $B$. We say that $f$ is *positive* if it preserves positive elements. We say that $f$ is *completely positive* if it is $n$-positive for every $n \in \mathbb{N}$, i.e. the map $M_n(f) : M_n(A) \to M_n(B)$ defined for every matrix $[x_{i,j}]_{1 \leq i,j \leq n} \in M_n(A)$ by $M_n(f)([x_{i,j}]_{1 \leq i,j \leq n}) = [f(x_{i,j})]_{1 \leq i,j \leq n}$ is positive. The map $f$ is called *multiplicative*, *involutive*, *unital* if it preserves multiplication, involution, and the unit, respectively. The map $f$ is called *subunital* whenever the inequalities $0 \leq f(1) \leq 1$ hold. A *state* on a C*-algebra $A$ is a completely positive unital map $s : A \to \mathbb{C}$.

Although W*-algebras are commonly defined in topological terms (as C*-algebras closed under several operator topologies) or equivalently in algebraic terms (as C*-algebras which are their own bicommutant), one can also equivalently define them in domain-theoretic terms [19], as we do next.

A completely positive map between C*-algebras is *normal* if its restriction to the unit interval is Scott-continuous [19, Proposition A.3]. A *W*-algebra* is a

C*-algebra $A$ such that the unit interval $[0,1]_A$ is a dcpo, and $A$ has a separating set of normal states: for every $x \in A^+$, if $x \neq 0$, then there is a normal state $s : A \to \mathbb{C}$ such that $s(x) \neq 0$ [25, Theorem III.3.16].

A linear map $f : A \to B$ between W*-algebras $A$ and $B$ is called an *NCPSU-map* if $f$ is normal, completely positive and subunital. The map $f$ is called an *NMIU-map* if $f$ is normal, multiplicative, involutive and unital. We note that every NMIU-map is necessarily an NCPSU-map and that W*-algebras are closed under formation of matrix algebras as in Example 10.

*Categorical Structure.* Let $\mathbf{W}^*_{\mathrm{NCPSU}}$ be the category of W*-algebras and NCPSU-maps and let $\mathbf{W}^*_{\mathrm{NMIU}}$ be its full-on-objects subcategory of NMIU-maps. Throughout the rest of the paper let $\mathbf{C} := (\mathbf{W}^*_{\mathrm{NCPSU}})^{\mathrm{op}}$ and let $\mathbf{V} := (\mathbf{W}^*_{\mathrm{NMIU}})^{\mathrm{op}}$. QPL types are interpreted as functors $[\![\Theta \vdash A]\!] : \mathbf{V}^{|\Theta|} \to \mathbf{V}$ and closed QPL types as objects $[\![A]\!] \in \mathrm{Ob}(\mathbf{V}) = \mathrm{Ob}(\mathbf{C})$. One should think of $\mathbf{V}$ as the category of *values*, because the interpretation of our values from §3 are indeed $\mathbf{V}$-morphisms. General QPL terms are interpreted as morphisms of $\mathbf{C}$, so one should think of $\mathbf{C}$ as the category of *computations*. We now describe the categorical structure of $\mathbf{V}$ and $\mathbf{C}$ and later we justify our choice for working in the opposite categories.

Both $\mathbf{C}$ and $\mathbf{V}$ have a symmetric monoidal structure when equipped with the spatial tensor product, denoted here by $(-\otimes-)$, and tensor unit $I := \mathbb{C}$ [11, Section 10]. Moreover, $\mathbf{V}$ is symmetric monoidal closed and also complete and cocomplete [11]. $\mathbf{C}$ and $\mathbf{V}$ have finite coproducts, given by direct sums of W*-algebras [2, Proposition 4.7.3]. The coproduct of objects $A$ and $B$ is denoted by $A + B$ and the coproduct injections are denoted $\mathrm{left}_{A,B} : A \to A + B$ and $\mathrm{right}_{A,B} : B \to A + B$. Given morphisms $f : A \to C$ and $g : B \to C$, we write $[f, g] : A + B \to C$ for the unique cocone morphism induced by the coproduct. Moreover, coproducts distribute over tensor products [2, §4.6]. More specifically, there exists a natural isomorphism $d_{A,B,C} : A \otimes (B + C) \to (A \otimes B) + (A \otimes C)$ which satisfies the usual coherence conditions. The initial object in $\mathbf{C}$ is moreover a zero object and is denoted 0. The W*-algebra of bits is $\mathbf{bit} := I + I = \mathbb{C} \oplus \mathbb{C}$.

The categories $\mathbf{V}, \mathbf{C}$ and $\mathbf{Set}$ are related by symmetric monoidal adjunctions:

$$\mathbf{Set} \underset{G}{\overset{F}{\rightleftarrows}} \mathbf{V} \underset{R}{\overset{J}{\rightleftarrows}} \mathbf{C} \qquad F \dashv G,\; J \dashv R \qquad [26,\text{ pp. } 11]$$

and the subcategory inclusion $J$ preserves coproducts and tensors up to equality.

Interpreting QPL within $\mathbf{C}$ and $\mathbf{V}$ is not an ad hoc trick. In physical terms, this corresponds to adopting the *Heisenberg picture* of quantum mechanics and this is usually done when working with infinite-dimensional W*-algebras (like we do). Semantically, this is necessary, because (1) our type system has conditional branching and we need to interpret QPL terms within a category with finite coproducts; (2) we have to be able to compute parameterised initial algebras to interpret inductive datatypes. The category $\mathbf{W}^*_{\mathrm{NCPSU}}$ has finite products, but it does *not* have coproducts, so by interpreting QPL terms within $\mathbf{C} = (\mathbf{W}^*_{\mathrm{NCPSU}})^{\mathrm{op}}$ we solve problem (1). For (2), the monoidal closure of $\mathbf{V} = (\mathbf{W}^*_{\mathrm{NMIU}})^{\mathrm{op}}$ is crucial, because it implies the tensor product preserves $\omega$-colimits.

| | | | |
|---|---|---|---|
| $\mathrm{tr} : M_n(\mathbb{C}) \to \mathbb{C}$ | $\mathrm{new}_\rho : \mathbb{C} \to M_{2^n}(\mathbb{C})$ | $\mathrm{meas} : M_2(\mathbb{C}) \to \mathbb{C} \oplus \mathbb{C}$ | $\mathrm{unitary}_S : M_{2^n}(\mathbb{C}) \to M_{2^n}(\mathbb{C})$ |
| $\mathrm{tr} :: A \mapsto \sum_i A_{i,i}$ | $\mathrm{new}_\rho :: a \mapsto a\rho$ | $\mathrm{meas} :: \begin{pmatrix} a & b \\ c & d \end{pmatrix} \mapsto \begin{pmatrix} a & d \end{pmatrix}$ | $\mathrm{unitary}_S :: A \mapsto SAS^\dagger$ |
| $\mathrm{tr}^\dagger : \mathbb{C} \to M_n(\mathbb{C})$ | $\mathrm{new}_\rho^\dagger : M_{2^n}(\mathbb{C}) \to \mathbb{C}$ | $\mathrm{meas}^\dagger : \mathbb{C} \oplus \mathbb{C} \to M_2(\mathbb{C})$ | $\mathrm{unitary}_S^\dagger : M_{2^n}(\mathbb{C}) \to M_{2^n}(\mathbb{C})$ |
| $\mathrm{tr}^\dagger :: a \mapsto aI_n$ | $\mathrm{new}_\rho^\dagger :: A \mapsto \mathrm{tr}(A\rho)$ | $\mathrm{meas}^\dagger :: \begin{pmatrix} a & d \end{pmatrix} \mapsto \begin{pmatrix} a & 0 \\ 0 & d \end{pmatrix}$ | $\mathrm{unitary}_S^\dagger :: A \mapsto S^\dagger AS$ |

Fig. 5: A selection of maps in the Schrödinger picture ($f : A \to B$) and their Hermitian adjoints ($f^\dagger : B \to A$) used in the Heisenberg picture.

*Convex Sums.* In both $\mathbf{C}$ and $\mathbf{W}^*_{\mathrm{NCPSU}}$, morphisms are closed under *convex sums*, which are defined pointwise, as usual. More specifically, given NCPSU-maps $f_1, \ldots, f_n : A \to B$ and real numbers $p_i \in [0, 1]$ with $\sum_i p_i \leq 1$, then the map $\sum_i p_i f_i : A \to B$ is also an NCPSU-map.

*Order-enrichment.* For W*-algebras $A$ and $B$, we define a partial order on $\mathbf{C}(A, B)$ by : $f \leq g$ iff $g - f$ is a completely positive map. Equipped with this order, our category $\mathbf{C}$ is $\mathbf{DCPO}_{\perp!}$-enriched [3, Theorem 4.3]. The least element in $\mathbf{C}(A, B)$ is also a zero morphism and is given by the map $\mathbf{0} : A \to B$, defined by $\mathbf{0}(x) = 0$. Also, the coproduct structure and the symmetric monoidal structure are both $\mathbf{DCPO}_{\perp!}$-enriched [2, Corollary 4.9.15] [3, Theorem 4.5].

*Quantum Operations.* For convenience, our operational semantics adopts the *Schrödinger picture* of quantum mechanics, which is the picture most experts in quantum computing are familiar with. However, as we have just explained, our denotational semantics has to adopt the Heisenberg picture. The two pictures are equivalent in finite dimensions and we will now show how to translate from one to the other. By doing so, we provide an explicit description (in both pictures) of the required quantum maps that we need to interpret QPL.

Consider the maps in Figure 5. The map *tr* is used to trace out (or discard) parts of quantum states. Density matrices $\rho$ are in 1-1 correspondence with the maps $new_\rho$, which we use in our semantics to describe (mixed) quantum states. The *meas* map simply measures a qubit in the computational basis and returns a bit as measurement outcome. The $unitary_S$ map is used for application of a unitary $S$. These maps work as described in the Schrödinger picture of quantum mechanics, i.e., the category $\mathbf{W}^*_{\mathrm{NCPSU}}$. For every map $f : A \to B$ among those mentioned, $f^\dagger : B \to A$ indicates its Hermitian adjoint [3]. In the Heisenberg picture, composition of maps is done in the opposite way, so we simply write $f^\ddagger := (f^\dagger)^{\mathrm{op}} \in \mathbf{C}(A, B)$ for the Hermitian adjoint of $f$ when seen as a morphism in $(\mathbf{W}^*_{\mathrm{NCPSU}})^{\mathrm{op}} = \mathbf{C}$. Thus, the mapping $(-)^\ddagger$ translates the above operations from the Schrödinger picture (the category $\mathbf{W}^*_{\mathrm{NCPSU}}$) to the Heisenberg picture (the category $\mathbf{C}$) of quantum mechanics.

[3] This adjoint exists, because $A$ and $B$ are *finite-dimensional* W*-algebras which therefore have the structure of a Hilbert space when equipped with the Hilbert-Schmidt inner product [27, pp. 145].

*Parameterised Initial Algebras.* In order to interpret inductive datatypes, we need to be able to compute parameterised initial algebras for the functors induced by our type expressions. $\mathbf{V}$ is ideal for this, because it is cocomplete and monoidal closed and so all type expressions induce functors on $\mathbf{V}$ which preserve $\omega$-colimits.

**Definition 11 (cf. [6, §6.1]).** *Given a category* $\mathbf{A}$ *and a functor* $T : \mathbf{A}^n \to \mathbf{A}$, *with* $n \geq 1$, *a* parameterised initial algebra *for* $T$ *is a pair* $(T^\sharp, \phi^T)$, *such that:*

- $T^\sharp : \mathbf{A}^{n-1} \to \mathbf{A}$ *is a functor;*
- $\phi^T : T \circ \langle Id, T^\sharp \rangle \Rightarrow T^\sharp : \mathbf{A}^{n-1} \to \mathbf{A}$ *is a natural isomorphism;*
- *For every* $A \in \mathrm{Ob}(\mathbf{A}^{n-1})$, *the pair* $(T^\sharp A, \phi^T_A)$ *is an initial* $T(A, -)$*-algebra.*

**Proposition 12.** *Every* $\omega$*-cocontinuous functor* $T : \mathbf{V}^n \to \mathbf{V}$ *has a parameterised initial algebra* $(T^\sharp, \phi^T)$ *with* $T^\sharp : \mathbf{V}^{n-1} \to \mathbf{V}$ *being* $\omega$*-cocontinuous.*

*Proof.* $\mathbf{V}$ is cocomplete, so this follows from [13, §4.3]. □

## 5 Denotational Semantics of QPL

In this section we describe the denotational semantics of QPL.

### 5.1 Interpretation of Types

The interpretation of a type $\Theta \vdash A$ is a functor $[\![\Theta \vdash A]\!] : \mathbf{V}^{|\Theta|} \to \mathbf{V}$, defined by induction on the derivation of $\Theta \vdash A$ in Figure 6. As usual, one has to prove this assignment is well-defined by showing the required initial algebras exist.

**Proposition 13.** *The assignment in Figure 6 is well-defined.*

*Proof.* By induction, every $[\![\Theta \vdash A]\!]$ is an $\omega$-cocontinuous functor and thus it has a parameterised initial algebra by Proposition 12. □

**Lemma 14 (Type Substitution).** *Given types* $\Theta, X \vdash A$ *and* $\Theta \vdash B$, *then:*

$$[\![\Theta \vdash A[B/X]]\!] = [\![\Theta, X \vdash A]\!] \circ \langle Id, [\![\Theta \vdash B]\!] \rangle.$$

*Proof.* Straightforward induction. □

For simplicity, the interpretation of terms is only defined on closed types and so we introduce more concise notation for them. For any closed type $\cdot \vdash A$ we write for convenience $[\![A]\!] := [\![\cdot \vdash A]\!](*) \in \mathrm{Ob}(\mathbf{V})$, where $*$ is the unique object of the terminal category $\mathbf{1}$. Notice also that $[\![A]\!] \in \mathrm{Ob}(\mathbf{C}) = \mathrm{Ob}(\mathbf{V})$.

**Definition 15.** *Given a closed type* $\cdot \vdash \mu X.A$, *we define an isomorphism (in* $\mathbf{V}$*):*

$$\mathrm{fold}_{\mu X.A} : [\![A[\mu X.A/X]]\!] = [\![X \vdash A]\!][\![\mu X.A]\!] \cong [\![\mu X.A]\!] : \mathrm{unfold}_{\mu X.A}$$

*where the equality is Lemma 14 and the iso is the initial algebra structure.*

*Example 16.* The interpretation of the types from Example 1 are $[\![\mathbf{Nat}]\!] = \bigoplus_{i=0}^{\omega} \mathbb{C}$ and $[\![\mathbf{List}(A)]\!] = \bigoplus_{i=0}^{\omega} [\![A]\!]^{\otimes i}$. Specifically, $[\![\mathbf{List}(\mathbf{qbit})]\!] = \bigoplus_{i=0}^{\omega} \mathbb{C}^{2^i \times 2^i}$.

$$\begin{aligned}
[\![\Theta \vdash A]\!] &: \mathbf{V}^{|\Theta|} \to \mathbf{V} \\
[\![\Theta \vdash \Theta_i]\!] &= \Pi_i \\
[\![\Theta \vdash I]\!] &= K_I \\
[\![\Theta \vdash \mathbf{qbit}]\!] &= K_{\mathbf{qbit}} \\
[\![\Theta \vdash A + B]\!] &= + \circ \langle [\![\Theta \vdash A]\!], [\![\Theta \vdash B]\!] \rangle \\
[\![\Theta \vdash A \otimes B]\!] &= \otimes \circ \langle [\![\Theta \vdash A]\!], [\![\Theta \vdash B]\!] \rangle \\
[\![\Theta \vdash \mu X.A]\!] &= [\![\Theta, X \vdash A]\!]^\sharp
\end{aligned}$$

Fig. 6: Interpretations of types. $K_A$ is the constant-$A$-functor.

$$\begin{aligned}
[\![\cdot \vdash * : I]\!] &\coloneqq \mathrm{id}_I \\
[\![\{n\} \vdash n : \mathbf{qbit}]\!] &\coloneqq \mathrm{id}_{\mathbf{qbit}} \\
[\![Q \vdash \mathbf{left}_{A,B} v : A + B]\!] &\coloneqq \mathrm{left} \circ [\![v]\!] \\
[\![Q \vdash \mathbf{right}_{A,B} v : A + B]\!] &\coloneqq \mathrm{right} \circ [\![v]\!] \\
[\![Q_1, Q_2 \vdash (v, w) : A \otimes B]\!] &\coloneqq [\![v]\!] \otimes [\![w]\!] \\
[\![Q \vdash \mathbf{fold}_{\mu X.A} v : \mu X.A]\!] &\coloneqq \mathrm{fold} \circ [\![v]\!]
\end{aligned}$$

Fig. 7: Interpretation of values.

$$\begin{aligned}
&[\![\Pi \vdash \langle \Gamma \rangle \ \mathbf{new\ unit}\ u \ \langle \Gamma, u : I \rangle]\!] \coloneqq \pi \mapsto r^{-1} \\
&[\![\Pi \vdash \langle \Gamma, x : A \rangle \ \mathbf{discard}\ x \ \langle \Gamma \rangle]\!] \coloneqq \pi \mapsto (r \circ (\mathrm{id} \otimes \diamond)) \\
&[\![\Pi \vdash \langle \Gamma, x : P \rangle \ y = \mathbf{copy}\ x \ \langle \Gamma, x : P, y : P \rangle]\!] \coloneqq \pi \mapsto (\mathrm{id} \otimes \triangle) \\
&[\![\Pi \vdash \langle \Gamma \rangle \ \mathbf{new\ qbit}\ q \ \langle \Gamma, q : \mathbf{qbit} \rangle]\!] \coloneqq \pi \mapsto \left( (\mathrm{id} \otimes \mathrm{new}^\ddagger_{|0\rangle\langle 0|}) \circ r^{-1} \right) \\
&[\![\Pi \vdash \langle \Gamma, q : \mathbf{qbit} \rangle \ b = \mathbf{measure}\ q \ \langle \Gamma, b : \mathbf{bit} \rangle]\!] \coloneqq \pi \mapsto \left( \mathrm{id} \otimes \mathrm{meas}^\ddagger \right) \\
&[\![\Pi \vdash \langle \Gamma, \vec{q} : \overrightarrow{\mathbf{qbit}} \rangle \ \vec{q} \mathrel{*}= S \ \langle \Gamma, \vec{q} : \overrightarrow{\mathbf{qbit}} \rangle]\!] \coloneqq \pi \mapsto \left( \mathrm{id} \otimes \mathrm{unitary}^\ddagger_S \right) \\
&[\![\Pi \vdash \langle \Gamma \rangle \ M; N \ \langle \Sigma \rangle]\!] \coloneqq \pi \mapsto ([\![N]\!](\pi) \circ [\![M]\!](\pi)) \\
&[\![\Pi \vdash \langle \Gamma \rangle \ \mathbf{skip} \ \langle \Gamma \rangle]\!] \coloneqq \pi \mapsto \mathrm{id} \\
&[\![\Pi \vdash \langle \Gamma, b : \mathbf{bit} \rangle \ \mathbf{while}\ b\ \mathbf{do}\ M \ \langle \Gamma, b : \mathbf{bit} \rangle]\!] \coloneqq \pi \mapsto \mathrm{lfp}(W_{[\![M]\!](\pi)}) \\
&[\![\Pi \vdash \langle \Gamma, x : A \rangle \ y = \mathbf{left}_{A,B}\ x \ \langle \Gamma, y : A + B \rangle]\!] \coloneqq \pi \mapsto (\mathrm{id} \otimes \mathrm{left}_{A,B}) \\
&[\![\Pi \vdash \langle \Gamma, x : B \rangle \ y = \mathbf{right}_{A,B}\ x \ \langle \Gamma, y : A + B \rangle]\!] \coloneqq \pi \mapsto \left( \mathrm{id} \otimes \mathrm{right}_{A,B} \right) \\
&[\![\Pi \vdash \langle \Gamma, y : A + B \rangle \ \mathbf{case}\ y\ \mathbf{of}\ \{\mathbf{left}\ x_1 \to M_1 \mid \mathbf{right}\ x_2 \to M_2\} \ \langle \Sigma \rangle]\!] \coloneqq \\
&\qquad \pi \mapsto ([[\![M_1]\!](\pi), [\![M_2]\!](\pi)] \circ d) \\
&[\![\Pi \vdash \langle \Gamma, x_1 : A, x_2 : B \rangle \ x = (x_1, x_2) \ \langle \Gamma, x : A \otimes B \rangle]\!] \coloneqq \pi \mapsto \mathrm{id} \\
&[\![\Pi \vdash \langle \Gamma, x : A \otimes B \rangle \ (x_1, x_2) = x \ \langle \Gamma, x_1 : A, x_2 : B \rangle]\!] \coloneqq \pi \mapsto \mathrm{id} \\
&[\![\Pi \vdash \langle \Gamma, x : A[\mu X.A/X] \rangle \ y = \mathbf{fold}\ x \ \langle \Gamma, y : \mu X.A \rangle]\!] \coloneqq \pi \mapsto (\mathrm{id} \otimes \mathrm{fold}) \\
&[\![\Pi \vdash \langle \Gamma, x : \mu X.A \rangle \ y = \mathbf{unfold}\ x \ \langle \Gamma, y : A[\mu X.A/X] \rangle]\!] \coloneqq \pi \mapsto (\mathrm{id} \otimes \mathrm{unfold}) \\
&[\![\Pi \vdash \langle \Gamma \rangle \ \mathbf{proc}\ f :: \ x : A \to y : B \ \{M\} \ \langle \Gamma \rangle]\!] \coloneqq \pi \mapsto \mathrm{id} \\
&[\![\Pi, f : A \to B \vdash \langle \Gamma, x : A \rangle \ y = f(x) \ \langle \Gamma, y : B \rangle]\!] \coloneqq (\pi, f) \mapsto (\mathrm{id} \otimes f)\,,
\end{aligned}$$

where $r$ is the right monoidal unit. For simplicity, we omit the monoidal associator.

Fig. 8: Interpretation of QPL terms.

#### 5.2 Copying and Discarding

Our type system is affine, so we have to construct discarding maps at all types. The tensor unit $I$ is a terminal object in $\mathbf{V}$ (but not in $\mathbf{C}$) which leads us to the next definition.

**Definition 17 (Discarding map).** *For any W*-algebra $A$, let $\diamond_A : A \to I$ be the unique morphism of $\mathbf{V}$ with the indicated domain and codomain.*

We will see that all values admit an interpretation as $\mathbf{V}$-morphisms and are therefore discardable. In physical terms, this means values are causal (in the sense mentioned in the introduction). Of course, this is not true for the interpretation of general terms (which correspond to $\mathbf{C}$-morphisms).

Our language is equipped with a copy operation on classical data, so we have to explain how to copy classical values. We do this by constructing a copy map defined at all *classical* types using results from [13,14].

**Proposition 18.** *Using the categorical data of* $\mathbf{Set} \underset{G}{\overset{F}{\rightleftarrows}} \mathbf{V}$ *(with $F \dashv G$), one can define a copy map $\triangle_{[\![P]\!]} : [\![P]\!] \to [\![P]\!] \otimes [\![P]\!]$ for every classical type $\cdot \vdash P$, such that the triple $\left([\![P]\!], \triangle_{[\![P]\!]}, \diamond_{[\![P]\!]}\right)$ forms a cocommutative comonoid in $\mathbf{V}$.*

We shall later see that the interpretations of our *classical* values are comonoid homomorphisms (w.r.t. Proposition 18) and therefore they may be copied.

#### 5.3 Interpretation of Terms

Given a variable context $\Gamma = x_1 : A_1, \ldots, x_n : A_n$, we interpet it as the object $[\![\Gamma]\!] := [\![A_1]\!] \otimes \cdots \otimes [\![A_n]\!] \in \mathrm{Ob}(\mathbf{C})$. The interpretation of a procedure context $\Pi = f_1 : A_1 \to B_1, \ldots, f_n : A_n \to B_n$ is defined to be the pointed dcpo $[\![\Pi]\!] := \mathbf{C}(A_1, B_1) \times \cdots \times \mathbf{C}(A_n, B_n)$. A term $\Pi \vdash \langle\Gamma\rangle\ M\ \langle\Sigma\rangle$ is interpreted as a Scott-continuous function $[\![\Pi \vdash \langle\Gamma\rangle\ M\ \langle\Sigma\rangle]\!] : [\![\Pi]\!] \to \mathbf{C}([\![\Gamma]\!], [\![\Sigma]\!])$ defined by induction on the derivation of $\Pi \vdash \langle\Gamma\rangle\ M\ \langle\Sigma\rangle$ in Figure 8. For brevity, we often write $[\![M]\!] := [\![\Pi \vdash \langle\Gamma\rangle\ M\ \langle\Sigma\rangle]\!]$, when the contexts are clear or unimportant.

We now explain some of the notation used in Figure 8. The rules for manipulating qubits use the morphisms $\mathrm{new}^{\ddagger}_{|0\rangle\langle 0|}$, $\mathrm{meas}^{\ddagger}$ and $\mathrm{unitary}^{\ddagger}_{S}$ which are defined in §4. For the interpretation of **while** loops, given an arbitrary morphism $f : A \otimes \mathbf{bit} \to A \otimes \mathbf{bit}$ of $\mathbf{C}$, we define a Scott-continuous endofunction

$$W_f : \mathbf{C}\,(A \otimes \mathbf{bit}, A \otimes \mathbf{bit}) \to \mathbf{C}(A \otimes \mathbf{bit}, A \otimes \mathbf{bit})$$
$$W_f(g) = \left[\mathrm{id} \otimes \mathrm{left}_{I,I},\ g \circ f \circ (\mathrm{id} \otimes \mathrm{right}_{I,I})\right] \circ d_{A,I,I},$$

where the isomorphism $d_{A,I,I} : A \otimes (I + I) \to (A \otimes I) + (A \otimes I)$ is explained in §4. For any pointed dcpo $D$ and Scott-continuous function $h : D \to D$, its *least fixpoint* is $\mathrm{lfp}(h) := \bigvee_{i=0}^{\infty} h^i(\bot)$, where $\bot$ is the least element of $D$.

*Remark 19.* The term semantics for defining and calling procedures does not involve any fixpoint computations. The required fixpoint computations are done when interpreting procedure stores, as we shall see next.

#### 5.4 Interpretation of Configurations

Before we may interpret program configurations, we first have to describe how to interpret values and procedure stores.

*Interpretation of Values.* A qubit pointer context $Q$ is interpreted as the object $[\![Q]\!] = \mathbf{qbit}^{\otimes |Q|}$. A value $Q \vdash v : A$ is interpreted as a morphism in $\mathbf{V}$ $[\![Q \vdash v : A]\!] : [\![Q]\!] \to [\![A]\!]$, which we abbreviate as $[\![v]\!]$ if $Q$ and $A$ are clear from context. It is defined by induction on the derivation of $Q \vdash v : A$ in Figure 7.

For the next theorem, recall that if $Q \vdash v : A$ is a classical value, then $Q = \cdot$.

**Theorem 20.** *Let $Q \vdash v : A$ be a value. Then:*

1. *$[\![v]\!]$ is discardable (i.e. causal). More specifically, $\diamond_{[\![A]\!]} \circ [\![v]\!] = \diamond_{[\![Q]\!]} = \mathrm{tr}^\ddagger$.*
2. *If $A$ is classical, then $[\![v]\!]$ is copyable, i.e., $\triangle_{[\![A]\!]} \circ [\![v]\!] = ([\![v]\!] \otimes [\![v]\!]) \circ \triangle_I$.*

We see that, as promised, interpretations of values may always be discarded and interpretations of classical values may also be copied. Next, we explain how to interpret value contexts. For a value context $Q; \Gamma \vdash V$, its interpretation is the morphism:

$$[\![Q; \Gamma \vdash V]\!] = \left( [\![Q]\!] \xrightarrow{\cong} [\![Q_1]\!] \otimes \cdots \otimes [\![Q_n]\!] \xrightarrow{[\![v_1]\!] \otimes \cdots \otimes [\![v_n]\!]} [\![\Gamma]\!] \right),$$

where $Q_i \vdash v_i : A_i$ is the splitting of $Q$ (see §3) and $[\![\Gamma]\!] = [\![A_1]\!] \otimes \cdots \otimes [\![A_n]\!]$. Some of the $Q_i$ can be empty and this is the reason why the definition depends on a coherent natural isomorphism. We write $[\![V]\!]$ as a shorthand for $[\![Q; \Gamma \vdash V]\!]$. Obviously, $[\![V]\!]$ is also causal thanks to Theorem 20.

*Interpretation of Procedure Stores.* The interpretation of a well-formed procedure store $\Pi \vdash \Omega$ is an element of $[\![\Pi]\!]$, i.e. a $|\Pi|$-tuple of morphisms from $\mathbf{C}$. It is defined by induction on $\Pi \vdash \Omega$ :

$$[\![\cdot \vdash \cdot]\!] = ()$$
$$[\![\Pi, f : A \to B \vdash \Omega, f :: x : A \to y : B\ \{M\}]\!] = ([\![\Omega]\!], \mathrm{lfp}([\![M]\!]([\![\Omega]\!], -))).$$

*Interpretation of Configurations.* Density matrices $\rho \in M_{2^n}(\mathbb{C})$ are in 1-1 correspondence with $\mathbf{W}^*_{\mathrm{NCPSU}}$-morphisms $\mathrm{new}_\rho : \mathbb{C} \to M_{2^n}(\mathbb{C})$ which are in turn in 1-1 correspondence with $\mathbf{C}$-morphisms $\mathrm{new}_\rho^\ddagger : I \to \mathbf{qbit}^{\otimes n}$. Using this observation, we can now define the interpretation of a configuration $\mathcal{C} = (M \mid V \mid \Omega \mid \rho)$ with $\Pi; \Gamma; \Sigma; Q \vdash (M \mid V \mid \Omega \mid \rho)$ to be the morphism

$$[\![\Pi; \Gamma; \Sigma; Q \vdash (M \mid V \mid \Omega \mid \rho)]\!] :=$$
$$\left( I \xrightarrow{\mathrm{new}_\rho^\ddagger} \mathbf{qbit}^{\otimes \dim(\rho)} \xrightarrow{[\![Q;\Gamma \vdash V]\!]} [\![\Gamma]\!] \xrightarrow{[\![\Pi \vdash \langle \Gamma \rangle\ M\ \langle \Sigma \rangle]\!]([\![\Pi \vdash \Omega]\!])} [\![\Sigma]\!] \right).$$

For brevity, we simply write $[\![(M \mid V \mid \Omega \mid \rho)]\!]$ or even just $[\![\mathcal{C}]\!]$ to refer to the above morphism.

#### 5.5 Soundness, Adequacy and Big-step Invariance

Since our operational semantics allows for branching, *soundness* is showing that the interpretation of configurations is equal to the sum of small-step reducts.

**Theorem 21 (Soundness).** *For any non-terminal configuration $\mathcal{C}$ :*

$$\llbracket \mathcal{C} \rrbracket = \sum_{\mathcal{C} \rightsquigarrow \mathcal{D}} \llbracket \mathcal{D} \rrbracket.$$

*Proof.* By induction on the shape of the term component of $\mathcal{C}$. □

*Remark 22.* The above sum and all sums that follow are well-defined convex sums of NCPSU-maps where the probability weights $p_i$ have been encoded in the density matrices.

A natural question to ask is whether $\llbracket \mathcal{C} \rrbracket$ is also equal to the (potentially infinite) sum of all terminal configurations that $\mathcal{C}$ reduces to. In other words, is the interpretation of configurations also invariant with respect to big-step reduction. This is indeed the case and proving this requires considerable effort.

**Theorem 23 (Big-step Invariance).** *For any configuration $\mathcal{C}$, we have:*

$$\llbracket \mathcal{C} \rrbracket = \bigvee_{n=0}^{\infty} \sum_{r \in \mathrm{TerSeq}_{\leq n}(\mathcal{C})} \llbracket \mathrm{End}(r) \rrbracket$$

The above theorem is the main result of our paper. This is a powerful result, because with big-step invariance in place, computational adequacy[4] at all types is now a simple consequence of the causal properties of our interpretation. Observe that for any configuration $\mathcal{C}$, we have a subunital map $\diamond \circ \llbracket \mathcal{C} \rrbracket : \mathbb{C} \to \mathbb{C}$ and evaluating it at 1 yields a real number $(\diamond \circ \llbracket \mathcal{C} \rrbracket)(1) \in [0, 1]$.

**Theorem 24 (Adequacy).** *For any normalised $\mathcal{C}$ : $(\diamond \circ \llbracket \mathcal{C} \rrbracket)(1) = \mathrm{Halt}(\mathcal{C})$.*

If $\mathcal{C}$ is not normalised, then adequacy can be recovered simply by normalising: $(\diamond \circ \llbracket \mathcal{C} \rrbracket)(1) = \mathrm{tr}(\mathcal{C})\mathrm{Halt}(\mathcal{C})$, for any possible configuration $\mathcal{C}$. The adequacy formulation of [17] and [5] is now a special case of our more general formulation.

**Corollary 25.** *Let $M$ be a closed program of unit type, i.e. $\cdot \vdash \langle \cdot \rangle\ M\ \langle \cdot \rangle$. Then:*

$$\llbracket (M \mid \cdot \mid \cdot \mid 1) \rrbracket (1) = \mathrm{Halt}(M \mid \cdot \mid \cdot \mid 1).$$

*Proof.* By Theorem 24 and because $\diamond_I = \mathrm{id}$. □

[4] Recall that a computational adequacy result has to establish an equivalent *purely denotational* characterisation of the operational notion of non-termination.

### 6 Conclusion and Related Work

There are many quantum programming languages described in the literature. For a survey see [7] and [16, pp. 129]. Some circuit programming languages (e.g. Proto-Quipper [21,22,15]), generate quantum circuits, but do not necessarily support executing quantum measurements. Here we focus on quantum languages which support measurement and which have either inductive datatypes or some computational adequacy result.

Our work is the first to present a detailed semantic treatment of user-defined inductive datatypes for quantum programming. In [17] and [5], the authors show how to interpret a quantum lambda calculus extended with a datatype for lists, but their syntax does not support any other inductive datatypes. These languages are equipped with lambda abstractions, whereas our language has only support for procedures. Lambda abstractions are modelled using constructions from quantitative semantics of linear logic in [17] and techniques from game semantics in [5]. We believe our model is simpler and certainly more physically natural, because we work only with mathematical structures used by physicists in their study of quantum mechanics. Both [17] and [5] prove an adequacy result for programs of unit type. In [20], the authors discuss potential categorical models for inductive datatypes in quantum programming, but there is no detailed semantic treatment provided and there is no adequacy result, because the language lacks recursion.

Other quantum programming languages without inductive datatypes, but which prove computational adequacy results include [9,12]. A model based on W*-algebras for a quantum lambda calculus without recursion or inductive datatypes was described in a recent manuscript [4]. In that model, it appears that currying is *not* a Scott-continuous operation, and if so, the addition of recursion renders the model neither sound, nor adequate. For this reason, we use procedures and not lambda abstractions in our language.

To conclude, we presented two novel results in quantum programming: (1) we provided a denotational semantics for a quantum programming language with inductive datatypes; (2) we proved that our denotational semantics is invariant with respect to big-step reduction. We also showed that the latter result is quite powerful by demonstrating how it immediately implies computational adequacy.

Our denotational model is based on W*-algebras, which are used by physicists to study quantum foundations. We hope this would make it useful for developing static analysis methods (based on abstract interpretation) that can be used for entanglement detection [18] and we plan on investigating this in future work.

**Acknowledgements.** We thank Andre Kornell, Bert Lindenhovius and Michael Mislove for discussions regarding this paper. We also thank the anonymous referees for their feedback. MR acknowledges financial support from the Quantum Software Consortium, under the Gravitation programme of the Dutch Research Council NWO. The remaining authors were supported by the French projects ANR-17-CE25-0009 SoftQPro, ANR-17-CE24-0035 VanQuTe and PIA-GDN/Quantex.
